# Key features of inhibitor binding to the human mitochondrial pyruvate carrier hetero-dimer

**DOI:** 10.1016/j.molmet.2022.101469

**Published:** 2022-03-10

**Authors:** Sotiria Tavoulari, Tom J.J. Schirris, Vasiliki Mavridou, Chancievan Thangaratnarajah, Martin S. King, Daniel T.D. Jones, Shujing Ding, Ian M. Fearnley, Edmund R.S. Kunji

**Affiliations:** Medical Research Council Mitochondrial Biology Unit, University of Cambridge, Keith Peters Building, Cambridge Biomedical Campus, Hills Road, Cambridge, CB2 0XY, United Kingdom

**Keywords:** Mitochondria, Mitochondrial transport, Mitochondrial pyruvate carrier, Inhibition, Small molecules

## Abstract

**Objective:**

The mitochondrial pyruvate carrier (MPC) has emerged as a promising drug target for metabolic disorders, including non-alcoholic steatohepatitis and diabetes, metabolically dependent cancers and neurodegenerative diseases. A range of structurally diverse small molecule inhibitors have been proposed, but the nature of their interaction with MPC is not understood, and the composition of the functional human MPC is still debated. The goal of this study was to characterise the human MPC protein *in vitro*, to understand the chemical features that determine binding of structurally diverse inhibitors and to develop novel higher affinity ones.

**Methods:**

We recombinantly expressed and purified human MPC hetero-complexes and studied their composition, transport and inhibitor binding properties by establishing *in vitro* transport assays, high throughput thermostability shift assays and pharmacophore modeling.

**Results:**

We determined that the functional unit of human MPC is a hetero-dimer. We compared all different classes of MPC inhibitors to find that three closely arranged hydrogen bond acceptors followed by an aromatic ring are shared characteristics of all inhibitors and represent the minimal requirement for high potency. We also demonstrated that high affinity binding is not attributed to covalent bond formation with MPC cysteines, as previously proposed. Following the basic pharmacophore properties, we identified 14 new inhibitors of MPC, one outperforming compound UK5099 by tenfold. Two are the commonly prescribed drugs entacapone and nitrofurantoin, suggesting an off-target mechanism associated with their adverse effects.

**Conclusions:**

This work defines the composition of human MPC and the essential MPC inhibitor characteristics. In combination with the functional assays we describe, this new understanding will accelerate the development of clinically relevant MPC modulators.

## Highlights

• Shared chemical properties defining inhibition of the human MPC hetero-dimer by structurally diverse inhibitors.
• High affinity binding to the MPC is not attributed to cysteine-mediated covalent bond formation.
• New high affinity inhibitors of the human MPC, with low nanomolar range potencies, are described.
• High throughput, protein-based *in vitro* assays for inhibitor screening to accelerate drug discovery efforts for MPC.

## Graphical abstract

**Figure:**
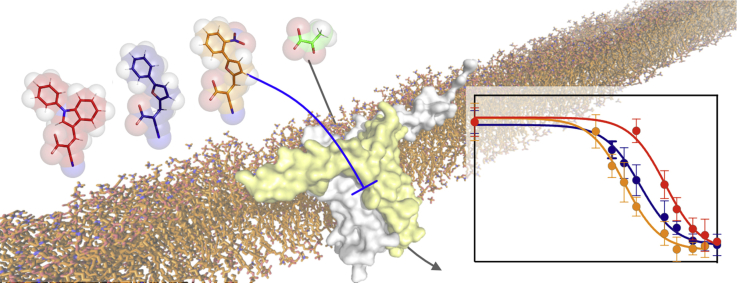

## 1 Introduction

The mitochondrial pyruvate carrier (MPC) is responsible for the import of pyruvate into the mitochondrial matrix where it enters the TCA cycle after its conversion to acetyl-CoA. This transport step links the cytosolic and mitochondrial energy metabolism, assigning to MPC a key role in the metabolic fate of the cell. In addition, MPC plays a pivotal role in several central metabolic pathways, such as gluconeogenesis, fatty acid and amino acid metabolism. Consequently, MPC is being investigated as a promising modulator of metabolic disorders, such as type-2 diabetes, non-alcoholic fatty liver disease (NAFLD) and non-alcoholic steatohepatitis (NASH) [[1], [2], [3], [4]]. It has also been proposed as a target for the treatment of neurodegenerative diseases [[5], [6], [7]] and metabolically dependent cancers [[8], [9], [10], [11]].

The mitochondrial pyruvate carrier is a protein complex formed by two small homologous membrane proteins [12,13]. In yeast, two alternative hetero-complexes form depending on the carbon source availability: Mpc1/Mpc2 in fermentative conditions and Mpc1/Mpc3 in respiratory conditions [14]. We recently reported the purification and functional reconstitution of the yeast MPCs and demonstrated that the functional unit is a hetero-dimer [15]. We have also shown that the individual yeast proteins can form homo-dimers in the absence of the other protomer, but they are inactive [15]. In humans, two proteins, MPC1 and MPC2, are ubiquitously expressed to form the MPC1/MPC2 complex, present in all tissues [16]. An additional complex can be formed in the testis, specifically in post meiotic germ cells [16]. This complex is formed by the same MPC2 protomer and MPC1L [16], which is homologous to MPC1 (Fig. S1). In separate studies, researchers have proposed that the individual human MPC2 protein is active for pyruvate transport as an autonomous entity [17], and the individual MPC1 and MPC2 protomers bind small molecule inhibitors in the absence of their partner protein [18]; this suggests fundamental differences in MPC functional complexes between yeast and humans. However, human MPC hetero-complexes have not yet been reconstituted into liposomes in a functional state, and their properties have not been determined or compared with homo-complexes.

A few different and often clinically relevant compounds have been proposed to inhibit pyruvate-driven respiration or pyruvate transport in cell-based systems or isolated mitochondria. The best characterised compounds for monitoring MPC inhibition are α-cyano-cinnamates and derivatives, such as the α-cyano-4-hydroxy-cinnamate (CHC) [19,20], or more potent analogues, such as the α-cyano-β-(1-phenylindol-3-yl)-acrylate, also known as UK5099 [21]; moreover, new UK5099 derivatives have recently been described [22]. Zaprinast, an inhibitor of cGMP-specific phosphodiesterase (PDE) and lead compound used for the development of sildenafil (Viagra), inhibits pyruvate-driven oxygen consumption in brain mitochondria and pyruvate uptake in liver mitochondria [23]. Silibinin, an adjunctive therapy in chronic hepatitis and cirrhosis, and valproic acid, a drug used to treat epilepsy, bipolar disorder and migraine headaches, have also been implicated in pyruvate uptake inhibition [24,25]. The anti-cancer agent lonidamine has been shown to inhibit pyruvate uptake in isolated mitochondria and to inhibit the monocarboxylate transporters (MCTs) with lower affinities [26,27]. Importantly, MPC is a target for thiazolidinediones (TZDs) [[28], [29], [30]], also known to exert their action via the peroxisome proliferator-activated receptor gamma (PPARγ) [31,32]. New generation TZDs, including MSDC-0160 (mitoglitazone) and MSDC-0602K, with decreased affinity for PPARγ have been developed [33] and are being considered in clinical trials for the treatment of non-alcoholic steatohepatitis [2] and Alzheimer's disease [34], emphasizing the importance of understanding the molecular nature of MPC inhibition.

In this study, we established *in vitro* systems relying on recombinant, heterologously expressed human MPC proteins to investigate the key features of inhibitor binding. First, we showed that the human MPC is a hetero-dimeric complex mediating pyruvate transport in proteoliposomes. Next, we compared structurally diverse compounds for binding and inhibitory potency and identified the critical and shared chemical properties important for MPC inhibition as well as new inhibitors with higher potency.

## 2 Materials and methods

### 2.1 Compounds

UK5099, CHC, zaprinast, lonidamine, mitoglitazone, pioglitazone, rosiglitazone, valproic acid, silibinin, entacapone and nitrofurantoin were purchased from Sigma Aldrich (Merck, Gillingham, UK). UK5099-based novel compounds (*i.e.,* compounds 1–13) were obtained from the Chembridge chemical store Hit2Lead (ChemBridge Corporation, CA, USA). All compounds were dissolved at a 100 mM stock concentration in DMSO.

### 2.2 Molecular biology

The codon-optimised gene sequences for human MPC1 (UniProt: Q9Y5U8), MPC2 (UniProt: O95563) and MPC1L (UniProt: P0DKB6) were synthesised (GenScript, Piscataway, NJ, USA) and cloned into the bidirectional expression vector pBEVY-GU (gift from Charles Miller; Addgene plasmid # 51,229 [35]). For expression of *mpc1* or *mpc1L*, the cDNA was subcloned into the EcoRI/SacI sites and for *mpc2* into the BamHI/XbaI sites. The sequence of MPC2 was designed to include a sequence coding for Factor Xa or TEV protease cleavage sites, followed by a tag of eight-histidines at their C-termini.

### 2.3 Protein expression and mitochondrial preparations

The expression plasmids were transformed into the *mpc* triple deletion strain of *Saccharomyces cerevisiae* SHY15 [12], kindly provided by Jean-Claude Martinou, using standard methods [36]. Successful transformants were selected on synthetic-complete uracil–dropout medium plates supplemented with 2% (*w/v*) glucose. Pre-cultures were grown in the same medium and used to inoculate 50 L of YPG medium containing 0.1% (*w/v*) glucose in an Applikon Pilot Plant 140 L bioreactor [37]. Protein expression was induced for 3 h with 0.4% (*w/v*) galactose, after 20 h of growth in YPG. Mitochondrial isolation was performed, as previously described [37], using a DYNO-MILL (Willy A. Bachofen AG, Basel, Switzerland). Isolated crude mitochondria were aliquoted, flash-frozen in liquid nitrogen and stored in −80 °C until use.

### 2.4 Affinity chromatography

The MPC1L/MPC2 and MPC1/MPC2 hetero-complexes were purified from mitochondria of the SHY15 strain expressing the relevant protein pairs. Immediately prior to purification, 1 g of mitochondria were thawed and suspended in buffer containing 20 mM Tris–HCl, pH 7.4, 150 mM NaCl, 10% (*v/v*) glycerol, one Complete EDTA-free protease inhibitor cocktail tablet (Roche, Basel, Switzerland) and 1% (*w/v*) lauryl maltose neopentyl glycol (LMNG, Anatrace, OH, USA). Mitochondria were solubilised for 1.5 h at 4 °C under gentle agitation and the insoluble material was removed by ultracentrifugation at 205,000×*g* for 45 min. The supernatant was incubated for 2 h with nickel Sepharose High Performance beads (Cytiva, MA, USA), in the presence of 10 mM imidazole then transferred into an empty column (Bio-Rad, Watford, UK). The column was first washed with 25 column volumes of buffer A (20 mM Tris–HCl, pH 7.4, 150 mM NaCl, 50 mM imidazole, 0.1% (*w/v*) LMNG, 0.1 mg/mL tetraoleoyl cardiolipin (TOCL, Avanti Polar Lipids, AL, USA)), followed by 20 column volumes of buffer B (20 mM Tris–HCl, pH 7.4, 150 mM NaCl, 0.1% (*w/v*) LMNG, 0.1 mg/mL TOCL). MPC1/MPC2 was eluted from the column by on-column digestion, in the presence of 5 mM CaCl_2_, for 12 h at 4 °C with 10 μg of Factor Xa protease (New England Biolabs, Hitchin, UK) per 1 g of mitochondria. MPC1L/MPC2 was eluted by on-column digestion for 12 h at 10 °C with 250 μg of MBP-TEV protease per 1 g of mitochondria. The mobile phase containing untagged MPC was separated from the resin with empty Proteus Midi spin columns (Generon, Slough, UK) at 200×*g* for 5 min. For MPC1L/MPC2, the TEV protease was removed by incubation of the purified protein with 250 μL amylose resin (New England Biolabs, Hitchin, UK). Protein concentrations were determined by the bicinchoninic acid assay (Thermo Fisher Scientific, Hemel, Hempstead, UK).

### 2.5 Size-exclusion chromatography

Analytical size-exclusion chromatography was performed on an ÄKTA Explorer (GE Healthcare, MA, USA) with a Superdex 200 10/300 GL column (GE Healthcare, MA, USA) equilibrated in SEC buffer (20 mM Tris–HCl, pH 7.4, 150 mM NaCl, 0.05% (w/v) LMNG, 0.05 mg/ml TOCL). Nickel purified MPC1L/MPC2 or Mpc1/Mpc3 were applied onto the column at 0.3 mL/min and 0.15 ml fractions were collected. The column was calibrated with a high molecular weight calibration kit (GE Healthcare, MA, USA) in the same buffer, without detergent and lipid.

### 2.6 Mass determination by SEC-MALLS

Aze-exclusion chromatography linked to multi-angle laser light sctteringSEC-MALLS analysis ) was performed with a Superdex 200 10/300 GL column (GE Healthcare, MA, USA) on an ÄKTA Explorer (GE Healthcare, MA, USA) coupled in-line with a light scattering detector (Dawn HELEOSII, Wyatt Technologies) and a refractometer (Optilab T-rREX, Wyatt Technologies). The MPC complexes were applied onto the Superdex 200 10/300 GL column equilibrated with 20 mM Tris–HCl, pH 7.4, 150 mM NaCl, 0.005% (w/v) LMNG, 0.005 mg/mL TOCL at 0.3 mL/min. All data were recorded and analysed with ASTRA 6.03 (Wyatt Technologies). Molecular weight calculations were performed using the protein-conjugate method [38] with the dn/dc value for protein of 0.185 mL/g and dn/dc value for LMNG-TOCL of 0.1675 mL/g [37]. The contributions of each protein to the overall protein–detergent–lipid complex were determined from the extinction coefficients *ε*_A280_, derived from the amino-acid sequence using the ProtParam tool on the ExPaSy server [39].

### 2.7 Thermostability shift analysis

The assessment of ligand binding was performed via shifts in protein thermostability upon binding using thermal denaturation on a rotary quantitative PCR (qPCR) instrument (Qiagen Rotor-Gene Q 2plex HRM, Venlo, Netherlands), as previously described [40]. In this method, cysteine residues, buried within the protein structure, become solvent-exposed during denaturation in a temperature ramp and then react with 7-diethylamino-3-(4-maleimidophenyl)-4-methylcoumarin (CPM) to form fluorescent-adducts. Briefly, a CPM working solution was prepared by diluting the CPM stock (5 mg/mL in dimethyl sulfoxide) 50-fold into assay buffer (20 mM Tris–HCl, pH 7.4, 150 mM NaCl, 0.1% (*w/v*) LMNG, 0.1 mg/mL TOCL) and incubated for 10 min at room temperature. In each analysis, performed in duplicates or triplicates, 3 μg of purified MPC was diluted into 45 μL of assay buffer containing the desired concentration of the inhibitor then incubated for 10 min on ice. Next, 5 μL of the CPM working solution were added. Samples were incubated on ice for an additional 10 min then subjected to a temperature ramp of 5.6 °C/min. The fluorescence increase was monitored with the HRM channel of the instrument (excitation at 440–480 nm, emission at 505–515 nm). Unfolding profiles were analysed with the Rotor-Gene Q software 2.3, and the peaks of their derivatives were used to determine the apparent melting temperature.

Ligand binding was also assessed using dye-free nano differential scanning fluorimetry (nano-DSF) to monitor fluorescence changes due to altered environments of tryptophan and tyrosine residues during unfolding. Protein samples in buffer containing 20 mM Tris–HCl pH 7.4, 150 mM NaCl, 0.1% (*w/v*) LMNG, 0.1 mg/mL TOCL, in the presence or absence of the indicated concentrations of small molecule inhibitors, were loaded into capillary tubes, and a temperature ramp of 5 °C/min was applied. The intrinsic fluorescence was measured using the NanoTemper Prometheus NT.48 (NanoTemper, München, Germany).

### 2.8 Reconstitution in proteoliposomes

Egg l-α-phosphatidylcholine 99% (*w/v*) (Avanti Polar Lipids, AL, USA) and TOCL were mixed in a 20:1 (*w/w*) ratio, dried under a stream of nitrogen and washed once with methanol before being dried again. Lipids were hydrated in 20 mM Tris–HCl, pH 8.0, 50 mM NaCl to a concentration of 12 mg/mL. Unlabeled pyruvate was added as a freshly made concentrated stock at a final concentration of 5 mM for internalisation. Lipids were solubilised with 1.2% (*v/v*) pentaethylene glycol monodecyl ether (Sigma) and freshly purified protein was added at a lipid-to-protein ratio of 250:1 (*w/w*). Samples were incubated on ice for 5 min, then liposomes were formed by the step-wise removal of pentaethylene glycol monodecyl ether by five additions at 20 min intervals of 60 mg Bio-Beads SM-2 (Bio-Rad, Watford, UK) with gentle mixing at 4 °C. A final addition of 480 mg Bio-Beads was incubated with the samples overnight. Proteoliposomes were first separated from the Bio-Beads by passage through empty spin columns (Bio-Rad, Watford, UK) and subsequently pelleted at 120,000×*g* for 60 min. The proteoliposomes were resuspended with a thin needle in 150 μL of their supernatant after the rest of the supernatant was removed.

### 2.9 Pyruvate transport assays

The time course of pyruvate homo-exchange was measured at room temperature. The transport was initiated by diluting proteoliposomes 200-fold into external buffer, 20 mM MES, pH 6.4, 50 mM NaCl, containing 50 μM [^14^C]-pyruvate (500,000 dpm, Perkin Elmer, MA, USA). The reaction (0–30 s) was terminated by rapid dilution into 8 volumes of ice-cold internal buffer (20 mM Tris–HCl, pH 8.0, 50 mM NaCl), followed by rapid filtration through cellulose nitrate 0.45 μm filters (Millipore, Gillingham, UK) and washing with an additional 8 volumes of buffer. The filters were dissolved in Ultima Gold scintillation liquid (Perkin Elmer, MA, USA) and the radioactivity was counted with a Perkin Elmer Tri-Carb 2800 RT liquid scintillation counter. For inhibition of pyruvate transport, various concentrations of compounds, as indicated in the figure legends, were simultaneously added to the liposomes with 50 μM radioactive substrate. The data analysis was performed with non-linear regression fittings using GraphPad Prism 7.0d ([Inhibitor] vs response, variable slope). The specific uptake rates were calculated based on the amount of protein used in reconstitutions, as estimated from bicinchoninic acid assay. The biological repeats represent independent proteoliposome preparations using protein from independent purifications.

### 2.10 Pharmacophore modeling

All molecules were prepared for pharmacophore modelling by setting the protonation at pH 7.4, which was also used for the thermostability assays, and by using Marvin Sketch (ChemAxon, Budapest, Hungary). Subsequently, pharmacophore models were generated using Ligandscout 4.3 Essential (Inte:ligand, Vienna, Austria). Three-dimensional structures were generated initially, and energy was minimised using the MMFF94 protocol (see Supplementary Table S2 for minimised energies). Secondly, conformations were generated using the iCon protocol, allowing a maximum of 200 configurations per molecule with an energy window of 20 kcal/mol and a root mean square (RMS) value of 0.8 (see Supplementary Table S2 for number of conformations generated). Next, maximally ten shared-feature pharmacophore models were generated for each individual molecule using the ‘pharmacophore fit’ scoring function. The coordinates of the pharmacophores were determined and used to calculate distances between features. To investigate pharmacophore features shared between different compounds, pharmacophore models were transferred to the screening interface. A screening library containing all compounds was constructed using the same conformation generation settings as described above, and a fit-score was calculated using the ‘pharmacophore fit’ scoring function.

### 2.11 Mass spectrometry

Different MPC inhibitors (Table1) or sulfhydryl reagents were first incubated with MPC1L/MPC2 in a folded state, then the protein molecular masses were measured by electrospray mass spectrometry. The MPC subunits (10 μg) were resolved by reverse-phase HPLC on a PLRP-S column (1.0 mm i.d. X 75 mm; VARIAN) using a gradient of 2-propanol in a solvent system compatible with the recovery of membrane proteins (Carroll et al., 2009). The eluate (50 μL/min) was introduced into a QTRAP4000 (SCIEX) mass spectrometer operating in positive ion single MS mode. The instrument was scanned from 700 m/z to 2300 m/z after calibration with ions from a mixture of 1.0 μM of myoglobin and 1.5 μM trypsinogen. Molecular masses were obtained by reconstruction of the spectra of multiply charged ions using PeakView (SCIEX).

**Table 1:** Molecular mass measurements of components of recombinant human MPC1L/MPC2 after reaction with either inhibitors or sulfhydryl modifiers.

Sample	Observed mass	Calc. mass^a^^,^^b^	Mass difference	Modifications e
**MPC protein - unliganded**				
MPC1L (5–136)	14647.3	14647.9	0.6	
MPC1L (2–136)	15005.5	15006.3	0.8	
MPC2 (2–137)	14984.0	14985.6	1.6	
**MPC + UK5099**				
MPC1L (5–136)	14647.3	14647.9	0.6	
MPC1L (2–136)	15005.7	15006.3	0.6	
MPC2 (2–127)	14983.9	14985.6	1.7	
**MPC + Zaprinast**				
MPC1L (5–136)	14647.6	14647.9	0.3	
MPC1L (2–136)	15005.9	15006.3	0.4	
MPC2 (2–137)	14984.1	14985.6	1.5	
**MPC + compound 7**				
MPC1L (5–136)	14647.4	14648.3	0.9	
MPC1L (2–136)	15005.8	15006.3	0.5	
MPC2 (2–137)	14984.0	14985.6	1.6	
**MPC + MTSEA**				
MPC1L (5–136)	14797.5	14648.3	+149.2	2 x MTSEA
MPC1L (2–136)	15155.6	15006.3	+149.3	2 x MTSEA
MPC2 (2–137)	15059.1	14985.6	+75	1 x MTSEA
**MPC + NEM**				
MPC2 (2–137)	15234.3 c	14985.6	+248.7	2 x NEM
MPC2 (2–137)	15108.5	14985.6	+122.9	1 x NEM
MPC2 (2–137)	15359.9	14985.6	+374.3	3 x NEM
MPC1L (5–136)	14648.1	14647.9	0.2	unmodified
MPC1L (5–136)	14772.4 c	14647.9	+124.1	1 x NEM
MPC1L (5–136)	14897.5 c	14647.9	+249.6	2 x NEM
MPC1L (5–136)	15023.4 d	14647.9	+374.9	3 x NEM
MPC1L (2–136)	15255.9 c	15006.3	+249.6	2 x NEM
MPC1L (2–136)	15130.6	15006.3	124.3	1 x NEM

a The calculated molecular mass of MPC2 assumes removal of C-terminal His Tag, removal of N-terminal Met and acetylation of the new N-terminal Alanine.

b The calculated masses of MPC1L include residues 2–136 or 5–136 after initiation of translation from either of two Methionines from an N-terminal sequence MARMAVLWRKMRDNF.

c Denote the major components in mass spectra of NEM modified protein.

d In the mass spectrum of MPC1L protein modified with NEM, the presence of any unmodified MPC1L (2–136) with mass of 15,006 Da is obscured.

e Modification by NEM results in a mass increase of 125.1 Da, and modification by MTSEA results in mass increase of 75.0 Da.

## 3 Results

### 3.1 Pyruvate transport and ligand binding by human MPC hetero-dimers

**Figure 1:**
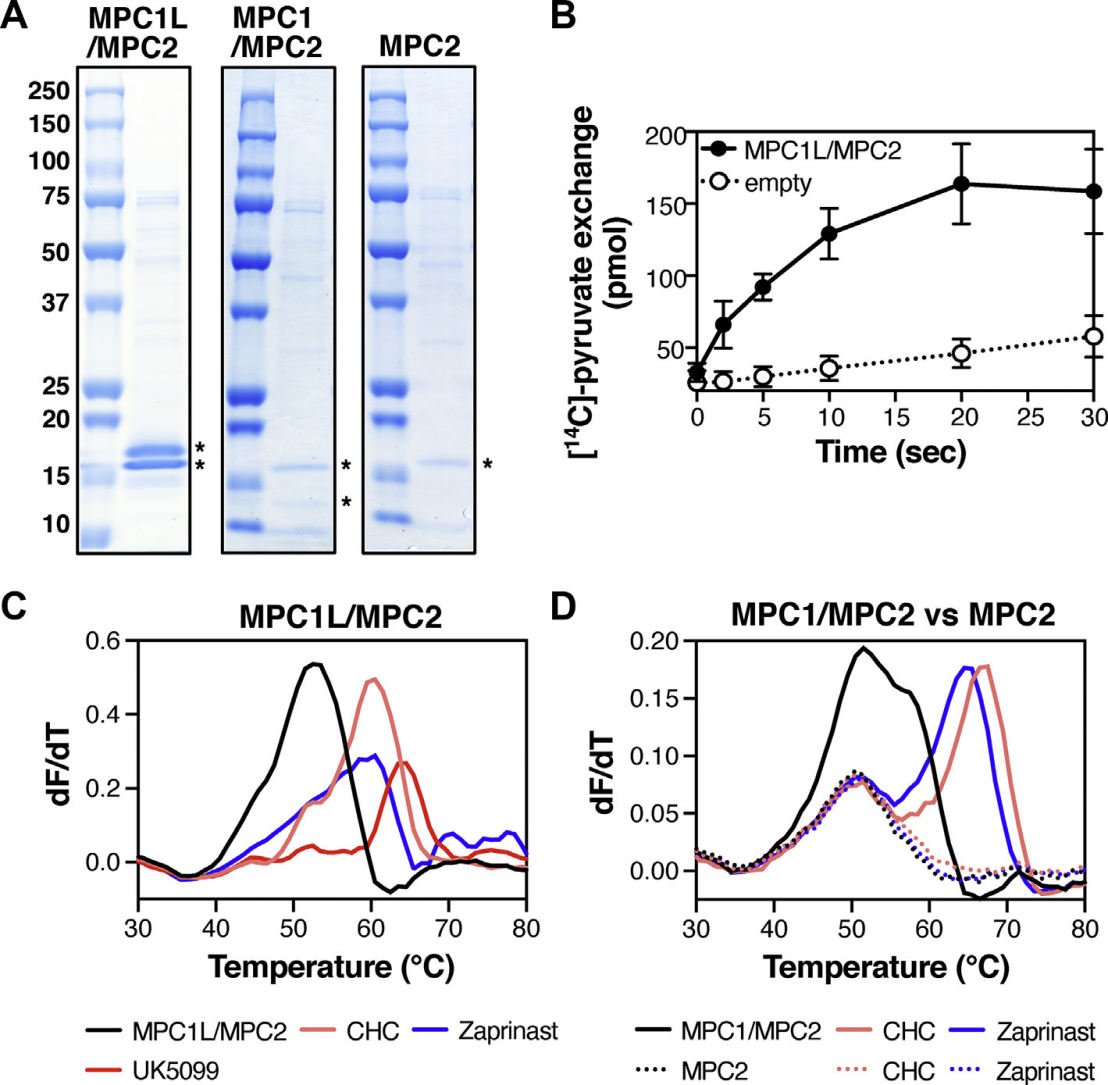
**Pyruvate transport and ligand binding by human MPC.** (**A**) Νickel affinity-purified MPC1L/MPC2 hetero-dimer (left), MPC1/MPC2 hetero-dimer (middle) and MPC2 protomer (right) analysed by SDS-PAGE and visualised by Coomassie Blue stain. MPC proteins are indicated by asterisks. (**B**) Time course of pyruvate homo-exchange at a ΔpH of 1.6 for the MPC1L/MPC2 hetero-dimer reconstituted into liposomes. (**C-D**) CPM thermostability shift assays for MPC hetero-dimers and individual MPC2 in the presence of absence of 100 μM MPC inhibitors. In (**B**), data represent the mean ± s.d. of six biological repeats, each performed with two technical replicates. In (**C–D)**, data are representative of two biological repeats, each performed with three or two technical replicates, respectively.

Human MPC hetero-complexes MPC1/MPC2 and MPC1L/MPC2 were expressed in the *S. cerevisiae mpc* triple deletion strain SHY15 [12] and purified by nickel-affinity chromatography using a cleavable eight-histidine tag at the C-terminus of MPC2; MPC1 or MPC1L remained untagged. From the two complexes tested, only MPC1L/MPC2 was purified with good yields (>0.7 mg of protein per gram of isolated mitochondria) and was characterised by an equimolar composition of the two protomers (Figure 1A, left), similar to the yeast Mpc1/Mpc3 hetero-dimer [15]. The size of the human MPC1L/MPC2 was also similar to the yeast Mpc1/Mpc3 [15] by size-exclusion chromatography in the same detergent/lipid mix (Fig. S2a), suggesting that it also forms a hetero-dimer. The calculated mass by size-exclusion chromatography linked to multi-angle laser light scattering (SEC-MALLS) (43 ± 6 kDa) along with the equimolar presence of the two protomers were also consistent with the formation of a hetero-dimer (Fig. S2b).

We then assessed the ability of the MPC1L/MPC2 dimer to transport pyruvate when reconstituted into liposomes (Figure 1B), as used for the yeast MPC [15]. The initial uptake rate of pyruvate transport was 0.87 ± 0.17 μmol/min/mg protein, similar to the rate previously measured for the yeast Mpc1/Mpc3 [15]. To evaluate ligand binding, we used thermostability shift assays. We monitored the unfolding of the protein population in a temperature ramp in the presence of 7-diethylamino-3-(4-maleimidophenyl)-4-methylcoumarin (CPM), which reacts with buried cysteine residues that become exposed due to denaturation (Figure 1C,D). In the presence of ligand, the protein population shifts to higher melting temperatures, due to the increased number of interactions forming [40]. A decrease in thermostability is also possible if a compound destabilises the complex. Unlike what we observed previously for the yeast protein [15], the thermostability results for the MPC1L/MPC2 hetero-dimer (Figure 1C) showed strong shifts in the presence of different MPC inhibitors; specifically, the melting temperature of the unliganded MPC1L/MPC2 shifted from 51.3 ± 0.2 ºC to 59.6 ± 0.4 ºC, 58.8 ± 0.4 ºC and 62.5 ± 0.9 ºC in the presence for the previously known inhibitors CHC, zaprinast and UK5099, respectively (Figure 1C and Supplementary Table S1). Overall, these results support a hetero-dimeric functional unit for the human complex, with high affinity for inhibitors.

We also purified the MPC1/MPC2 complex, albeit with low yields (<0.1 mg of protein per gram of isolated crude mitochondria) and a ratio of MPC1 to MPC2 of approximately 1:2, suggesting excess of MPC2 during co-expression and/or co-purification (Figure 1A, middle). In thermostability shift assays for MPC1/MPC2, we observed strong shifts in the presence of CHC and Zaprinast consistent with high affinity inhibitor binding in this complex. However, in both the unliganded and liganded forms, the protein presented two main peaks, one of which did not shift in the temperature ramp. We then attempted to individually express and purify the protomers of this complex to dissect the unfolding profile of MPC1/MPC2. Although no MPC1 protein could be purified alone, we could purify MPC2 alone, a protein previously proposed to form functional homo-complexes [17]. The individually purified MPC2 was only produced in low yields (Figure 1A, right) and could not be tested for transport activity. We were, however, able to test it for inhibitor binding in thermostability shift assays, where neither CHC nor zaprinast showed any shift (Figure 1D). Additionally, the MPC2 unfolding curve aligned with the first peak of MPC1/MPC2 which does not shift in the presence of inhibitors. The results indicate that binding of inhibitors to MPC2 homomers, also included in the MPC1/MPC2 sample, is absent or very weak.

### 3.2 Shared chemical features enabling MPC inhibition by structurally distinct molecules

To understand inhibitor binding by the variety of structurally diverse small molecules proposed, we performed the first comparative analysis of inhibitory potency using functionally reconstituted purified MPC.

**Figure 2:**
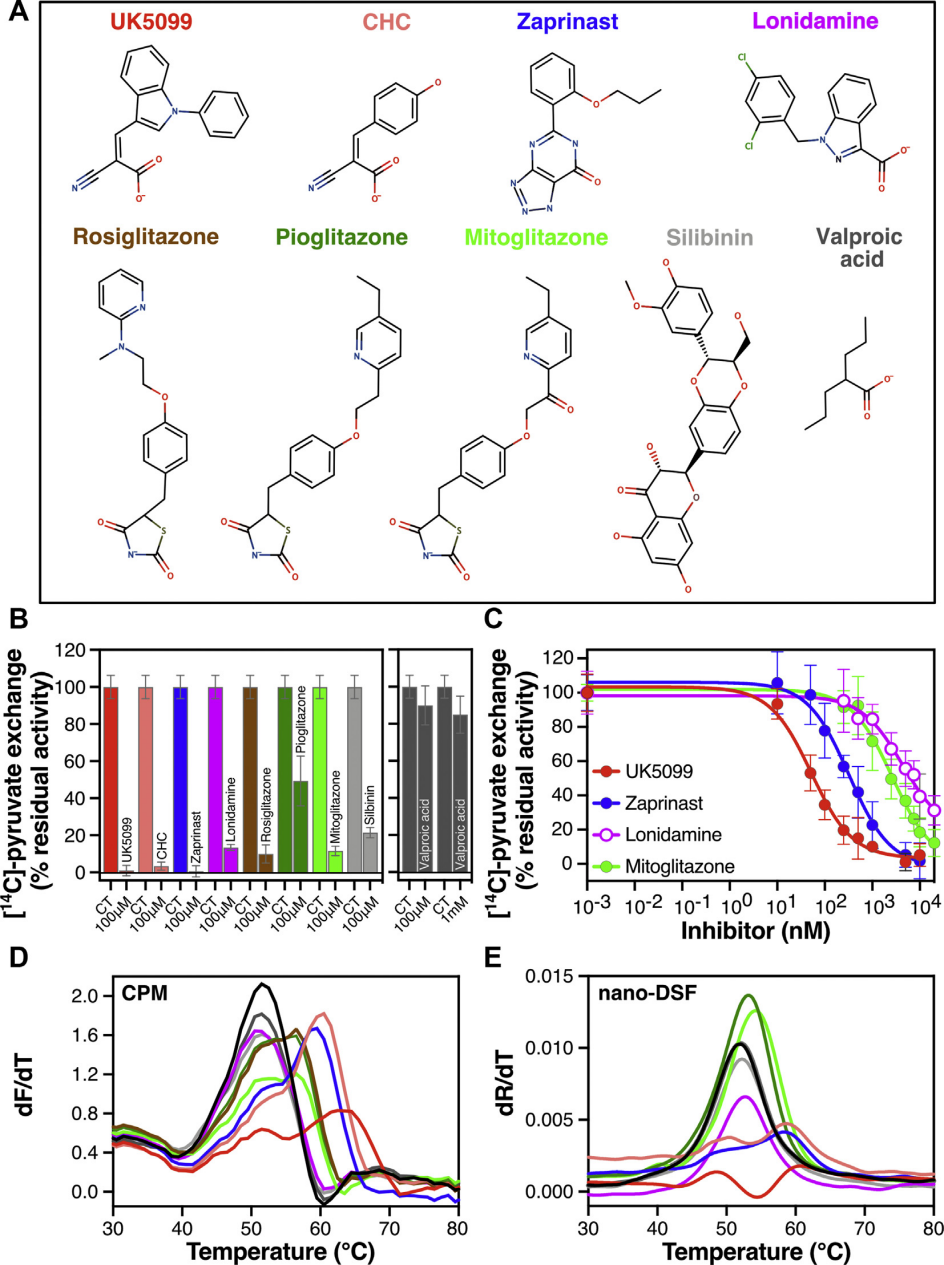
**Comparative analyses of ligand binding and pyruvate transport inhibition by structurally distinct small molecules.** (**A**) Chemical structures of previously claimed MPC inhibitors. Color coding is consistent across all panels. (**B**) The compounds listed in (A) were tested for inhibition of pyruvate transport on MPC1L/MPC2 proteoliposomes at 100 μM, and the remaining transport activity was compared to that in their absence (CT). (**C**) Inhibition of [^14^C]-pyruvate homo-exchange by UK5099 (10–10,000 nM), zaprinast (10–10,000 nM), lonidamine (250–20,000 nM) and mitoglitazone (250–20,000 nM). (**D**) First derivative of MPC1L/MPC2 unfolding profiles with or without compounds (100 μM) via the CPM thermostability assay. (**E**) First derivative of MPC1L/MPC2 unfolding profiles (330 nm/350 nm ratio) with or without compounds (100 μM) via nano-DSF. In (**B**), data points represent the mean ± s.d. of two biological repeats performed in triplicate. In (**C**), data points represent the mean ± s.d of four (UK5099) or three (zaprinast, lonidamine, mitoglitazone) biological repeats, each performed in triplicate. Results in (**D–E**) represent characteristic experiments repeated independently. Averaged data from different biological repeats are summarised in Table S1.

First, we tested all the listed molecules (Figure 2A), representative of different classes of MPC inhibitors, for their ability to inhibit pyruvate transport by MPC1L/MPC2 at a high concentration of 100 μM (Figure 2B). UK5099, CHC and zaprinast completely inhibited pyruvate transport at this concentration, whereas lonidamine, the TZDs and silibinin inhibited to a lower extent, ranging from 10%–50% of residual activity compared to control conditions. In contrast, the effect of valproic acid on pyruvate transport was minimal even at 1 mM final concentration (Figure 2B, right panel). To confirm that UK5099, CHC and zaprinast have a higher inhibitory potency than the TZDs, lonidamine or silibinin, we determined IC_50_-values of pyruvate transport inhibition for prototypic MPC inhibitors, as shown in Figure 2C. Indeed, UK5099 showed the highest inhibitory potency followed by zaprinast, with IC_50_-values of 52.6 ± 8.3 nM and 321 ± 42 nM, respectively. By contrast, lonidamine and mitoglitazone had lower potencies, with IC_50_-values in the low micromolar range (4.6 ± 1.3 μM and 2.7 ± 0.8 μM, respectively). Interestingly, UK5099 inhibits human MPC1L/MPC2 with two hundred times higher potency compared to the yeast protein. Zaprinast, lonidamine and the TZDs also have much higher potencies for the human protein [15], suggesting key differences in the binding site between the two species.

Despite exceptional reliability, the pyruvate transport assay did not constitute a high throughput solution for compound screening. Therefore, we evaluated thermostability shift as a primary screening approach for MPC binders. We tested all compounds listed in Figure 2A for their effect on MPC thermostability using two thermostability shift assays: the CPM assay (Figure 2D) and nano-differential scanning fluorimetry (nano-DSF), which relies on the intrinsic fluorescence of tryptophan and tyrosine residues (Figure 2E). Both assays yielded similar results for the unfolding of the unliganded dimer with apparent melting temperature values at 51.3 ± 0.2 ºC and 51.9 ± 0.1 ºC by the CPM and nano-DSF assay, respectively. Incubation of the dimer with each inhibitor at the same concentration of 100 μM resulted in a range of thermal shifts (Table S1). In the CPM assay, UK5099 yielded the highest thermal shift of all compounds (11.2 ± 1.1 ºC compared to the no-ligand control), followed by CHC (8.3 ± 0.6 ºC) and zaprinast (7.5 ± 0.6 ºC), consistent with the transport assay data showing highest inhibitory potencies for these compounds. The TZDs yielded a more moderate shift (up to 5 ºC) and valproic acid had no effect at all, also consistent with transport. No thermal shifts were observed for lonidamine and silibinin at a concentration of 100 μM in the CPM assay (Figure 2D and Table S1) or by nano-DSF (Figure 2E and Table S1), despite inhibiting pyruvate transport at the same concentration. However, at 1 mM, a positive thermal shift in the CPM assay was observed for silibinin, and a negative thermal shift was observed for lonidamine (Fig. S3).

Comparisons with the CPM assay showed that a few compounds suffered from fluorescence quenching in nano-DSF. UK5099, CHC and zaprinast shifted the denaturation curve from 51.9 ± 0.1 ºC to 61.1 ± 0.6 ºC, 58.9 ± 0.4 ºC and 58.2 ± 0.3 ºC, respectively (Figure 2E). Pioglitazone and mitoglitazone resulted in smaller thermal shifts compared to the CPM analysis, reaching Tm values of 53.0 ± 0.2 ºC and 53.8 ± 0.4 ºC, respectively, and rosiglitazone could not be tested due to autofluorescence. Due to these interferences, the CPM thermostability shift assay was chosen for subsequent investigations of the binding properties. However, data from both CPM and nano-DSF were consistent with the idea that UK5099, CHC and zaprinast are the strongest binders.

We then compared the macromolecular interaction characteristics of the prototypic MPC inhibitors UK5099, zaprinast, lonidamine and mitoglitazone and of the substrate pyruvate. A pharmacophore model was generated by structural alignment and resulted in a representation of all shared pharmacophore features (Figs. S4a and g). Interestingly, all inhibitors share three closely spaced (< 2.95 Å) hydrogen bond acceptor groups (Figure 4C–G, groups III, IV, V), also present in the substrate pyruvate (Figure 4A,B and G), suggesting that the inhibitors bind into the MPC substrate-binding pocket in a similar way as pyruvate. All prototypic inhibitors additionally share a central hydrophobic aromatic ring (Figure 4C–G, groups I and II), that is not present in pyruvate. Another pharmacophore feature shared by some, but not all inhibitors, consists of a negatively charged ionizable group (*i.e.*, in lonidamine, UK5099, and mitoglitazone) (Figs. S4B–G, group VI). However, the presence of this particular feature did not cluster with higher or lower affinity inhibitors.

### 3.3 Compound evolution increases the inhibitory potency and highlights the key chemical features

Next, we aimed to understand and enhance the inhibitory potency using the shared pharmacophore features (Fig. S4) of the known MPC inhibitors as a starting point. Because UK5099 showed the highest inhibitory potency (Figure 2), while sharing the three–hydrogen bond acceptors (Fig. S4g), we chose the carboxyl and cyanide functional groups of UK5099 as a molecular template to search for new chemical entities. We explored over 1,120,000 small molecules (*i.e.,* Chembridge Express and Core libraries) for compounds that contain this molecular template as well as an adjacent central aromatic ring.

**Figure 3:**
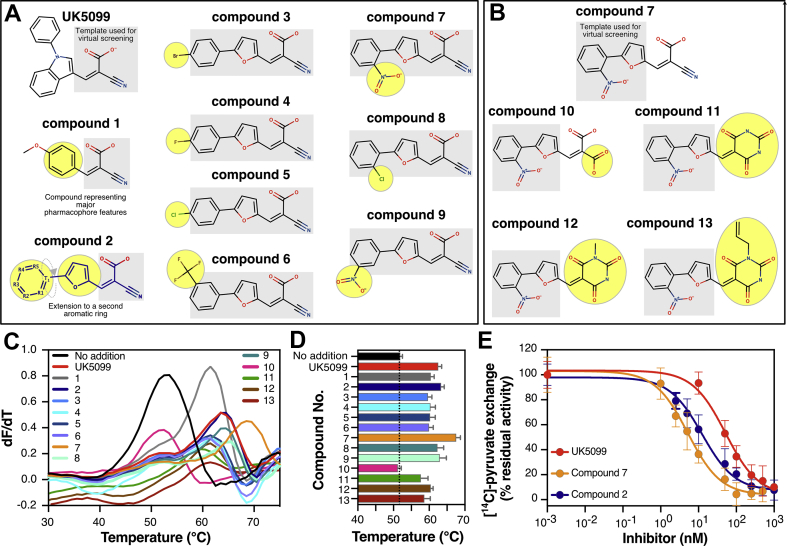
**Chemical evolution of MPC inhibitors.** (**A**) Chemical structures selected using the three hydrogen bond acceptors of UK5099. The template used for virtual screening is highlighted in a grey box. The chemical groups added or changed in each compound are highlighted in yellow. (**B**) Chemical structures of compounds where the cyanide and carboxylic acid groups have been replaced in the background of the structure of compound 7. The template used for virtual screening is highlighted in a grey box. The chemical groups added or changed in each compound are highlighted in yellow. (**C**) First derivatives obtained from CPM thermostability shift assays for the compounds in (**A**) and (**B**). (**D**) The Tm values, calculated from the first derivative in thermostability shift assays, were plotted in a bar graph. The dotted line indicates the melting temperature of unliganded MPC. Color coding is as in (**C**). (**E**) Inhibition of [^14^C]-pyruvate homo-exchange by UK5099 (10–10,000 nM), compounds 2 (2.5–1,000 nM) and 7 (1–500 nM). In (**C**), data are representative of three biological repeats. Data in (**D**) have been calculated from three independent biological repeats (values included in Table S1). In (**E**), data points represent the mean ± s.d of three biological repeats, each performed in triplicate. The averaged IC_50_-values were 52.6 ± 8.3 nM, 13.5 ± 4.1 nM and 5.4 ± 1.1 nM for UK5099, compound 2 and 7, respectively.

Compound 1 (Figure 3A), which embodies all the main features of our pharmacophore, was a good MPC binder as it yielded a thermostability shift to 60.4 ± 1.0 °C from 51.3 ± 0.6 °C in the absence of the ligand (Figure 3C,D and Table S1). This result demonstrated the validity of the pharmacophore model (Fig. S4g) and the importance of these features for MPC binding. An extension in the region following the central aromatic ring moiety (compound 2, Figure 3A), with an additional aromatic ring, increased the thermostability to 63.4 ± 0.9 °C, more than compound 1 or UK5099 (Figure 3C,D and Table S1). Indeed, as demonstrated by pyruvate transport inhibition in proteoliposomes, compound 2 is a more potent inhibitor than UK5099, with an IC_50_-value at 12.4 ± 4.6 nM, compared to 52.6 ± 8.3 nM for UK5099 (Figure 3E).

To investigate the second aromatic ring further, we used the general chemical formula of compound 2 (Figure 3A, highlighted in blue) as a starting point and selected compounds with additional chemical groups on this aromatic ring. Notably, R_1_ and R_2_ are equivalent to R_5_ and R_4_, respectively, due to a molecular torsion point (T_1_). Introduction of hydrophilic groups at the R_3_ position, as shown in compounds 3, 4 and 5 decreased binding compared to compound 2 and UK5099 (Figure 3C,D and Table S1). However, in the case of compound 7, binding was enhanced by a nitro group in position R_1_ (or R_5_) (Figure 3A), which shifted the MPC thermostability to 67.8 ± 1.2 °C, more than the thermal shifts of compound 2 or UK5099 (Tm; compound 2: 63.4 ± 0.9 °C UK5099: 62.6 ± 0.9 °C) (Figure 3C,D and Table S1). Compound 7 inhibited pyruvate transport in proteoliposomes, with an IC_50_-value of 5.4 ± 1.1 nM, approximately ten times lower than that of UK5099 (52.6 ± 8.3 nM) and two times lower than that of compound 2 (12.4 ± 4.6 nM) (Figure 3E), confirming an important role of the nitro group in position R_1_ (or R_5_) for binding.

To investigate whether the reactive nature of the nitro group in compound 7 contributes to its higher potency, we tested compound 8, in which a chloride group replaces the nitro group. This led to a thermostability shift lower than that of compound 7 (Figure 3A, C-Dand Table S1). Compound 9, in which the nitro group is relocated to position R_2_ (or R_4_), decreased the thermal shift compared to compound 7 but retained a thermal shift similar to that of compound 2 (Figure 3A,C and Dand Table S1). Replacement of the nitro group at position R_2_ (or R_4_) in compound 6 by a less reactive group reduced the thermal shift compared to compound 9 (Figure 3B–D). Therefore, the higher thermal shift elicited by compound 7 is possibly due to additional interactions introduced by the nitro group [41].

We evaluated the importance of the hydrogen bond acceptors provided by cyanide and carboxylic groups in compound 7 by testing compounds where these moieties are replaced by others (Figure 3B). First, we tested the replacement of the cyanide group by a carboxyl group (*i.e.,* compound 10). No thermal shift could be detected by compound 10 (Figure 3C–D and Table S1), indicating that the interaction with MPC was abolished or very weak. This observation is in accordance with earlier observations that showed a disruption of inhibitory capacity upon replacement of the cyanide group of UK5099 [21]. This result could be explained by the introduction of a negatively charged and larger moiety at the position of the hydrogen bond acceptor (feature III, Figure 3G). None of the other inhibitors have a negative group in that position (Figs. S4c–f and Figure 3A–B). The inability of compound 10 to produce any thermostability shift despite the presence of all other chemical features of compound 7, including the activated double bond, emphasises the importance of the hydrogen bond acceptors for binding to MPC.

Subsequently, we tested compounds that partly match the pyruvate-like pharmacophore. Both the cyanide and carboxyl group were replaced by a barbituric acid (compound 11), which matches with two of the three hydrogen bond acceptor features, by aligning with the ketone group (feature III) and carboxylic acid group (feature IV) of pyruvate (Fig. S5). We observed binding to MPC by this compound, albeit with smaller thermal shifts compared to UK5099, and by two closely related compounds (*i.e.*, compound 12 and 13), which have a slightly larger functional group (Figure 3C–D and Table S1). This result demonstrates that matching two of the hydrogen acceptor features might be a minimal requirement for binding to MPC.

### 3.4 High affinity inhibition is not attributed to covalent interactions with the human MPC

We investigated whether UK5099 and compound 7, which share inhibitory potencies in the low nanomolar range, form covalent interactions with MPC. A common characteristic of these compounds is an activated double bond, a putative Michael addition site. It has been hypothesised that UK5099 could inhibit MPC by using Michael addition to form a covalent bond with the thiol group of a cysteine [21,42,43]. Cys54 in MPC2 has been proposed to engage with an irreversible MPC chemical probe [42], raising the question whether the high affinity inhibitors with activated double bonds also react covalently with this cysteine.

Two approaches were used to investigate the potential covalent interactions of MPC with UK5099 and derivatives. First, the molecular masses of unliganded human MPC1L/MPC2 were measured by electrospray mass spectrometry and compared with the same protein complex after inhibition with UK5099, compound 7 or zaprinast. The first two inhibitor compounds contain the activated double bond, and zaprinast lacks it. Covalent modification of MPC by an inhibitor would increase the molecular mass of either MPC protomer, but the mass spectra of all MPC complexes were identical in the presence or absence of inhibitors, and no molecular mass differences in MPC protomers were observed (Table 1). However, when the thiol reactive compounds NEM or MTSEA were used as positive controls, both MPC subunits were modified covalently, and mass differences associated with modification of cysteine residues were observed (Table 1). These results demonstrate clearly that the inhibitor interactions with MPC are not covalent.

**Figure 4:**
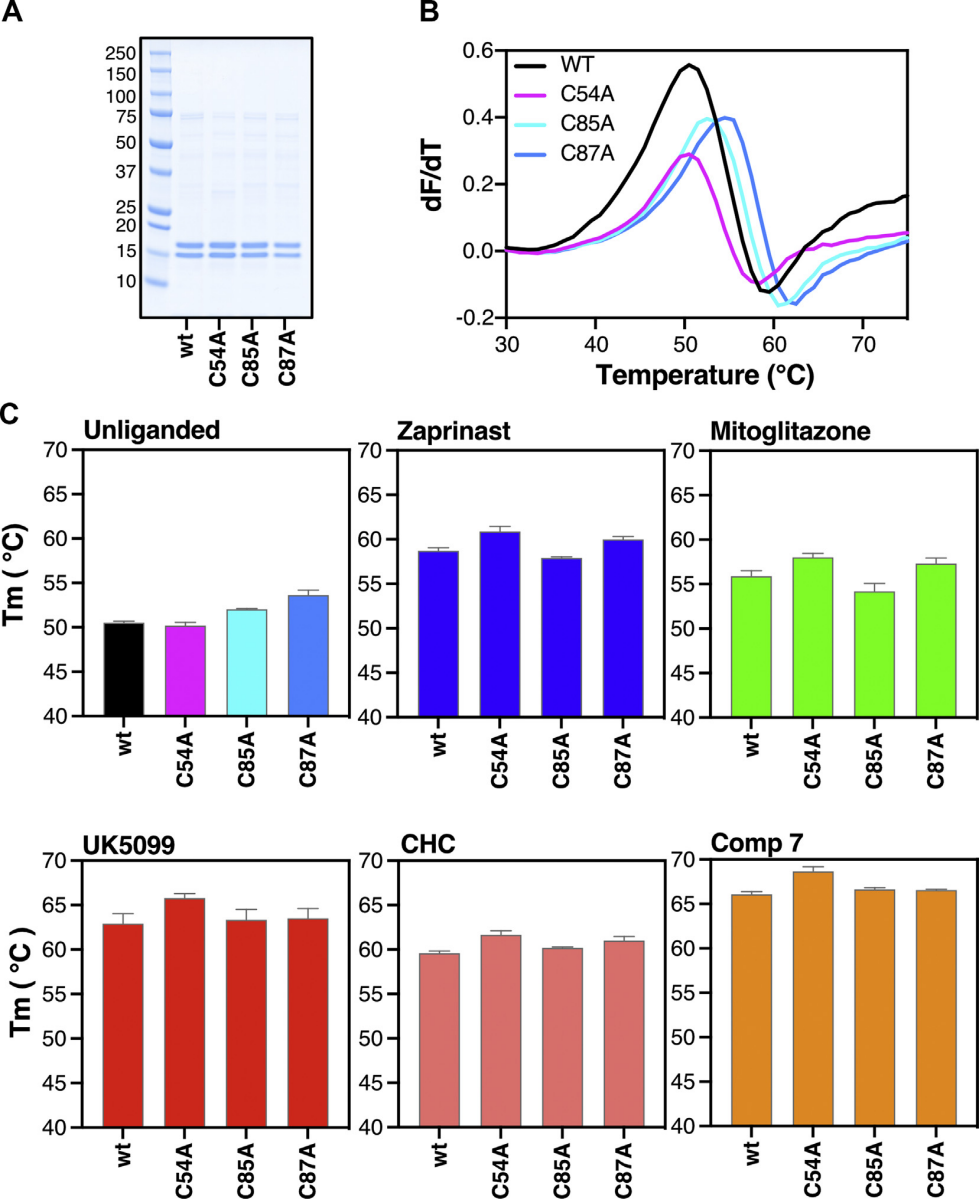
**MPC cysteines do not form covalent interactions with inhibitors.** (**A**) Cysteine-to-alanine replacement mutants purified in detergent in parallel with wild type (wt) human MPC1L/MPC2 via nickel affinity chromatography. (**B**) First derivative of protein unfolding profiles for wild type and cysteine-to-alanine replacement mutants via CPM thermostability assay. (**C**) CPM thermostability for wild-type and cysteine-to-alanine replacement mutants in the absence or presence of zaprinast, and mitoglitazone, which do not feature the activated double bond, or CHC, UK5099 and compound 7, each containing an activated double bond. In (**B**) data are representative of two biological repeats and three technical repeats. In (**C**), data represent the mean ± s.d of two biological repeats and three technical repeats.

Secondly, we mutated each cysteine of MPC2 (Cys54) or MPC1L (Cys85 or Cys87) separately to alanine. Figure 4A–B shows the purification and unfolding profiles of the three cysteine-to-alanine mutants, demonstrating that each was folded in detergent/lipid-containing buffer. We reasoned that if the low nanomolar potencies of UK5099 or compound 7 are due to covalent bond formation, then abolishing such a critical interaction by mutation of an interacting cysteine to alanine should translate to a broad decrease in binding and therefore to a decrease in the observed thermostability shifts in the presence of inhibitor. We compared wild type and cysteine-to-alanine mutants for the thermostability shift elicited by UK5099, CHC and compound 7, representing compounds with an activated double bond (Figure 4C). We also tested zaprinast and the TZD mitoglitazone, representing compounds without the activated double bond. For each of these mutants, we observed thermostability shifts comparable to wild type with all tested compounds (Figure 4C). Interestingly, mutating Cys54 to alanine in MPC2 did not reduce, but rather increased, the thermostability shifts, which is incompatible with this residue participating in any critical interaction with these inhibitors. This result indicates an indirect, positive effect on binding by reducing the size of the side chain. The thermostability shift was also not abolished when each of the cysteines in MPC1L were mutated to alanine. Taken together, these results challenge previous proposals for cysteine-mediated covalent bonds between MPC and specific inhibitors.

### 3.5 An extended pharmacophore model successfully identifies commonly used drugs as potent MPC inhibitors

**Figure 5:**
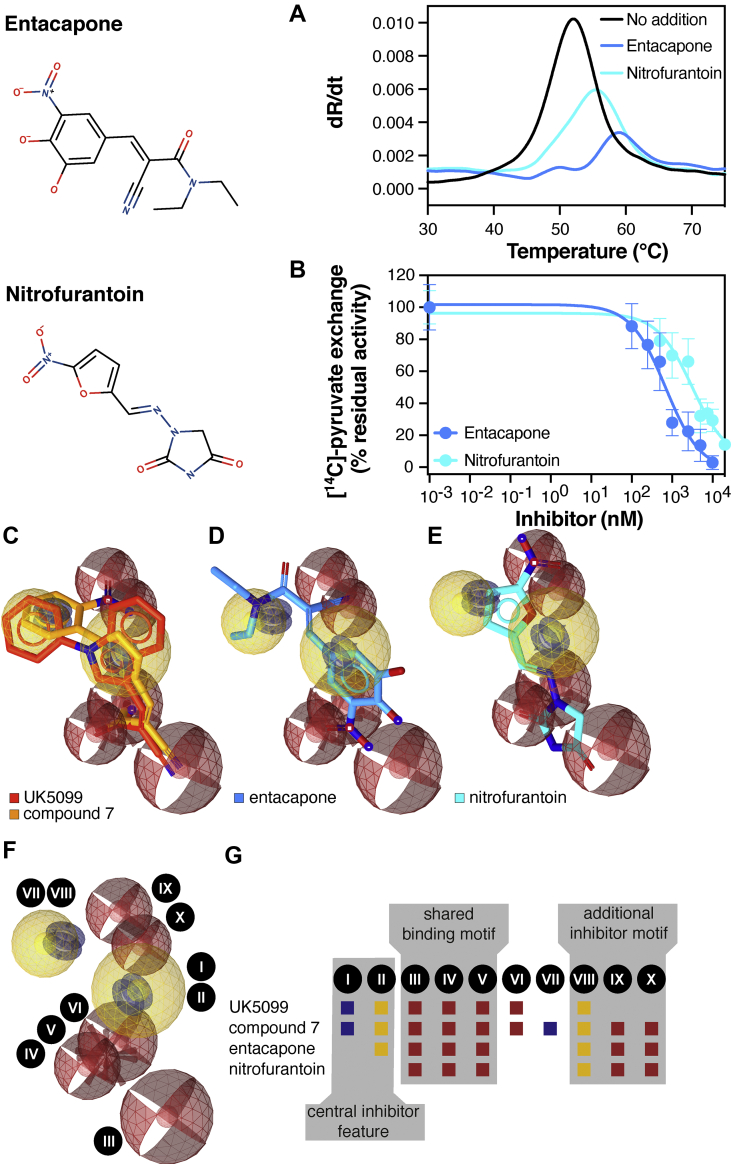
**Matching of an extended pharmacophore model predicts MPC inhibition.** (**A**) Thermostability assays for nitrofurantoin and entacapone using nano-DSF. (**B**) Inhibition of [^14^C]-pyruvate homo-exchange by entacapone (100–10,000 nM) and nitrofurantoin (500–20,000 nM). (**C**) Pharmacophore features of compound 7 and comparison with UK5099. Red spheres indicate hydrogen acceptor features, yellow spheres indicate hydrophobic ring features, red star indicate negative ionizable features and bleu tori indicate aromatic ring features. (**D**) Fitting of entacapone in the extended pharmacophore. (**E**) Fitting of nitrofurantoin in the extended pharmacophore. (**F-G**) 3D and graphical representation of the extended pharmacophore with the identified features (Roman numberals) and their presence in the various compounds. The pharmacophore features are as follows: I and VII; aromatic ring (blue boxes), II and VIII; hydrophobic ring (yellow boxes), III, IV, V, IX and X; hydrogen bond acceptors (maroon boxes). In (**A-B**), data are representative of two biological repeats, each performed in triplicate.

Having established the essential features for MPC inhibition, we tested whether their presence could be used to identify new compounds that inhibit MPC. We created a refined pharmacophore model where we incorporated all features resulting in the increased affinity of compound 7 (Figure 5C,G), including an additional aromatic structure (feature VIII) and two hydrogen bond acceptors (features IX and X). Importantly, both aromatic rings of UK5099 overlap with the ones in compound 7, which may be contributing to the high potency of these inhibitors (Figure 5C,G and Fig. S6).

We asked whether this model can be used to identify off-target effects of existing small molecule drugs on MPC. Several medicines in development or in clinical practice suffer from mitochondrial toxicity, but their mitochondrial targets are usually unknown. In this way, we have identified two commonly used compounds as potent MPC inhibitors, reported previously to interfere with mitochondrial function [[44], [45], [46]]. The first one, entacapone ((2*E*)-2-cyano-3-(3,4-dihydroxy-5-nitrophenyl)-*N*,*N*-diethylprop-2-enamide), is used in combination therapy for Parkinson's disease; the other, nitrofurantoin ((*E*)-1-[(5-nitro-2-furyl)methylideneamino]imidazolidine-2,4-dione), is an antibiotic used to treat bladder infections. Interestingly, the pharmacophore features of both drugs fit the essential pyruvate-like binding motif as well as the additional binding motif, similar to compound 7 (Figure 5D–G and Fig. S6).

Both compounds were tested on purified MPC1L/MPC2 using nano-DSF (Figure 5A), as they both had quenching effects on CPM fluorescence. Interestingly, entacapone, which contains all the essential features of the extended pharmacophore, shifted the MPC unfolding curve from 51.9 ± 0.1 °C to 58.2 ± 1.0 °C, whilst a more moderate shift to 54.7 ± 0.2 °C was observed with nitrofurantoin (Figure 5A and Table S1). Consistent with these results, the inhibitory potency of entacapone in MPC1L/MPC2 proteoliposomes was 630 ± 53 nM, whereas the IC_50_ value for nitrofurantoin was in the low micromolar range (3.3 ± 2.6 μM). The decreased potency of nitrofurantoin compared to entacapone could be explained by the absence of the central aromatic ring moiety (Figure 5F,G). Therefore, the proposed key chemical features for MPC inhibition can guide the identification of new or previously unidentified small molecule inhibitors.

## 4 Discussion

The molecular identification of MPC has sparked an interest to explore the potential of this key metabolic protein as a drug target; in the absence of structural information, however, our understanding of pyruvate transport and inhibition remains limited. Different claims have been made regarding the ability of MPC homo- and hetero-complexes to support transport and binding [[15], [16], [17], [18],^47^]. We have demonstrated the formation of the two proposed human hetero-dimers by co-expressing and co-purifying tagged MPC2 with untagged MPC1 or MPC1L, and we have reconstituted the human hetero-dimer MPC1L/MPC2 into functional liposomes, providing evidence that hetero-dimer formation mediates robust pyruvate transport activity. This result is consistent with our previous study of the yeast MPC complexes [15] and studies by others showing that expression of both MPC proteins is necessary for pyruvate transport in mitochondria [12,13,47].

We showed that both hetero-dimers bind known MPC inhibitors, whereas MPC1 alone could not be purified, and MPC2 did not bind small molecules without its counterpart. This notion contradicts previous reports claiming binding of inhibitors to MPC2 homomers [17,18]. Notably, the binding affinities of known inhibitors to MPC1 or MPC2 homomers that others have proposed were in the micromolar range [18], whereas studies in isolated mitochondria had predicted affinities in the low nanomolar range for mammalian MPCs (*i.e.,* K_i_ for UK5099 was ∼50 nM in isolated mitochondria [21]). Consistent with the studies in mitochondria, we report inhibitory potencies in the low nanomolar range for the hetero-dimer (*i.e.,* IC_50_-value for UK5099 is 52.6 ± 8.3 nM in our study). We propose that maximal binding of inhibitors and substrate occurs in the interface between protomers. Similar to the yeast homo-dimers [15], MPC2 should be unable to transport pyruvate and fails to form a functional binding pocket, as the inhibitor study shows. Moreover, an asymmetric substrate such as pyruvate requires an asymmetric binding pocket for co-ordination that cannot be provided by a homomer.

It is an interesting observation that the MPC1L/MPC2 hetero-dimer, expressed in testis, binds to and is inhibited by compounds (Figure 2) previously identified for their ability to inhibit the ubiquitously expressed MPC1/MPC2 hetero-dimer in human cells. This observation supports the idea that the two hetero-dimers should share a similar binding site, in agreement with their high sequence conservation (Fig. S1). On the contrary, the inhibitory potencies of the tested compounds were 25–200 times higher for the human MPC1L/MPC2 compared to the yeast Mpc1/Mpc3, suggesting important differences in inhibitor co-ordination between species.

From the inhibitors tested in our system, UK5099 was clearly the one with the highest potency of all previously reported compounds. The second most potent inhibitor was zaprinast, followed by the anticancer agent lonidamine and the insulin sensitizer mitoglitazone. Consistent with our work, lonidamine inhibits MPC in isolated mitochondria with a K_i_ at 2.5 μM [26]. The high affinity of α-cyano-cinnamates and derivatives for MPC has been previously attributed to an activated double bond, proposed but never shown, to form Michael addition adducts with cysteine residues in MPC. This idea was developed based on the altered absorption spectra of double bond containing compounds upon addition of β-mercaptoethanol in solution [43,48]. However, these experiments were performed on the small molecule compounds alone, not in the presence of protein. Additionally, MPC labelled by these compounds could not be detected on SDS-PAGE. As explanation, it was proposed that the covalent modification of MPC is reversible, but no direct proof of this hypothesis has ever been provided. More recently, Cys54 in MPC2 was found to engage with an irreversible chemical probe that is chemically unrelated to the cinnamates or other MPC inhibitors [42]. It was hypothesised that UK5099 could be engaging with this same cysteine, but again, the covalent binding of UK5099 was not shown. A covalent interaction could explain the high potencies of UK5099 and derivatives relative to other MPC inhibitors, but we show here that this is not the case. No cysteine modifications were observed by ESI-MS molecular mass measurements of MPC after inhibition. NEM, a Michael acceptor used as a control, reacts with cysteine residues in MPC via Michael additions, and they could be readily detected in our mass spectrometry analysis. Replacement of Cys54 of MPC2 to alanine did not prevent, but rather increased, binding of different inhibitors tested. Replacement of each of the cysteines in MPC1L also resulted in similar thermostability compared to wild-type in the presence of any inhibitor. Therefore, UK5099 and derivatives do not bind to MPC covalently, and the irreversibility observed in cellular studies [29,49] can therefore be attributed solely to high affinity binding.

Understanding the common pharmacophore properties of MPC inhibitors allowed us to identify more MPC binders and improve the inhibitory potency. Our pharmacophore analysis revealed that substrate and small molecule inhibitors share an arrangement of three hydrogen bond acceptors. In the substrate, the hydrogen bond acceptors are provided by carboxyl and ketone groups. In agreement, MPC was also implicated in the transport of the ketone bodies acetoacetate and β-hydroxybutyric acid [50,51]. In inhibitors, the three hydrogen bond acceptors can be supplied by a carboxyl group and a nitrile group, as in UK5099 and compounds 1 to 9. Consistent with the importance of the carboxyl and nitrile groups as efficient hydrogen bond acceptors, α-fluorocinnamate is 1,000 times less potent than α-cyano-cinnamate [21].

Distinct between inhibitors and substrates, but common amongst all inhibitors, is a central aromatic ring moiety that appears to be the second requirement for MPC inhibition, after the hydrogen bond acceptors. A second aromatic ring moiety is an additional feature that might be adding to the number of interactions between inhibitor and MPC to increase affinity. Moreover, addition of a nitro group to this second moiety led to a molecule with increased inhibitory potency (compound 7). These new features allowed us to understand binding of two commonly used drugs entacapone and nitrofurantoin and could guide the identification of additional off-target effects on MPC.

We have described the development of protein-based *in vitro* assays for MPC and have shown the use of a thermostability shift assay as a primary screening high-throughput approach allowing high affinity MPC binders to be identified. In combination with a proteoliposome-based transport assay, thermostability shift assays have allowed us to identify new small molecule inhibitors and extend the potency of previously known ones to lower nanomolar range. By combining *in vitro* and *in silico* analyses, we have mapped the pharmacophore properties of MPC binders and propose that a set of three hydrogen bond acceptors followed by a hydrophobic moiety are minimal requirements for high affinity binding, whereas covalent bonds are not formed between MPC and its inhibitors. This knowledge could be key to accelerating efforts in the development of clinically relevant MPC modulators.

## Data availability

Source data have been uploaded to the Dryad repository (https://doi.org/10.5061/dryad.stqjq2c2x)

## Contributions

Conception and design of the research: S.T., T.J.J.S., and E.R.S.K., Molecular Biology: S.T., D.T.D.J., M.S.K., Biochemical and Biophysical data acquisition: S.T., V.M., C.T., Pharmacophore modelling: T.J.J.S., Mass Spectrometry: S.D., and I.M.F., Data analysis: S.T. and T.J.J.S., Data interpretation: S.T., T.J.J.S., I.M.F, and E.R.S.K., Writing of the paper: S.T., T.J.J.S. and E.R.S.K. All authors read and revised the paper.
