# Supplementary material for: Key features of inhibitor binding to the human mitochondrial pyruvate carrier hetero-dimer
Source: Mol Metab. 2022 Mar 10;60:101469. doi: 10.1016/j.molmet.2022.101469 (PMC8968063; doi:10.1016/j.molmet.2022.101469)
Supplement: Multimedia component 1 [file mmc1.docx]

**SUPPLEMENTARY INFORMATION**

**Title:** Key features of inhibitor binding to the human mitochondrial pyruvate carrier hetero-dimer

**Authors:** Sotiria Tavoulari, Tom J.J. Schirris, Vasiliki Mavridou, Chancievan Thangaratnarajah, Martin S. King, Daniel T.D. Jones, Shujing Ding, Ian M. Fearnley and Edmund R.S. Kunji

**Figure S1. Sequence alignment and secondary structure prediction of human MPC proteins**

The alignment was generated by Clustal Omega [1], followed by manual curation. The aligned residues are colored by the ZAPPO color scheme in which aliphatic, polar, aromatic, positively charged, negatively charged, Pro/Gly and Cys, are colored pink, green, orange, blue, red, magenta and yellow, respectively. The asterisks indicate identical residues and the colon conserved substitutions. Also indicated are putative transmembrane helices, loop regions and the N-terminal amphipathic helix. The secondary structure elements were assigned based on PSIPRED [2], MEMSAT3 [3] and conservation analysis.


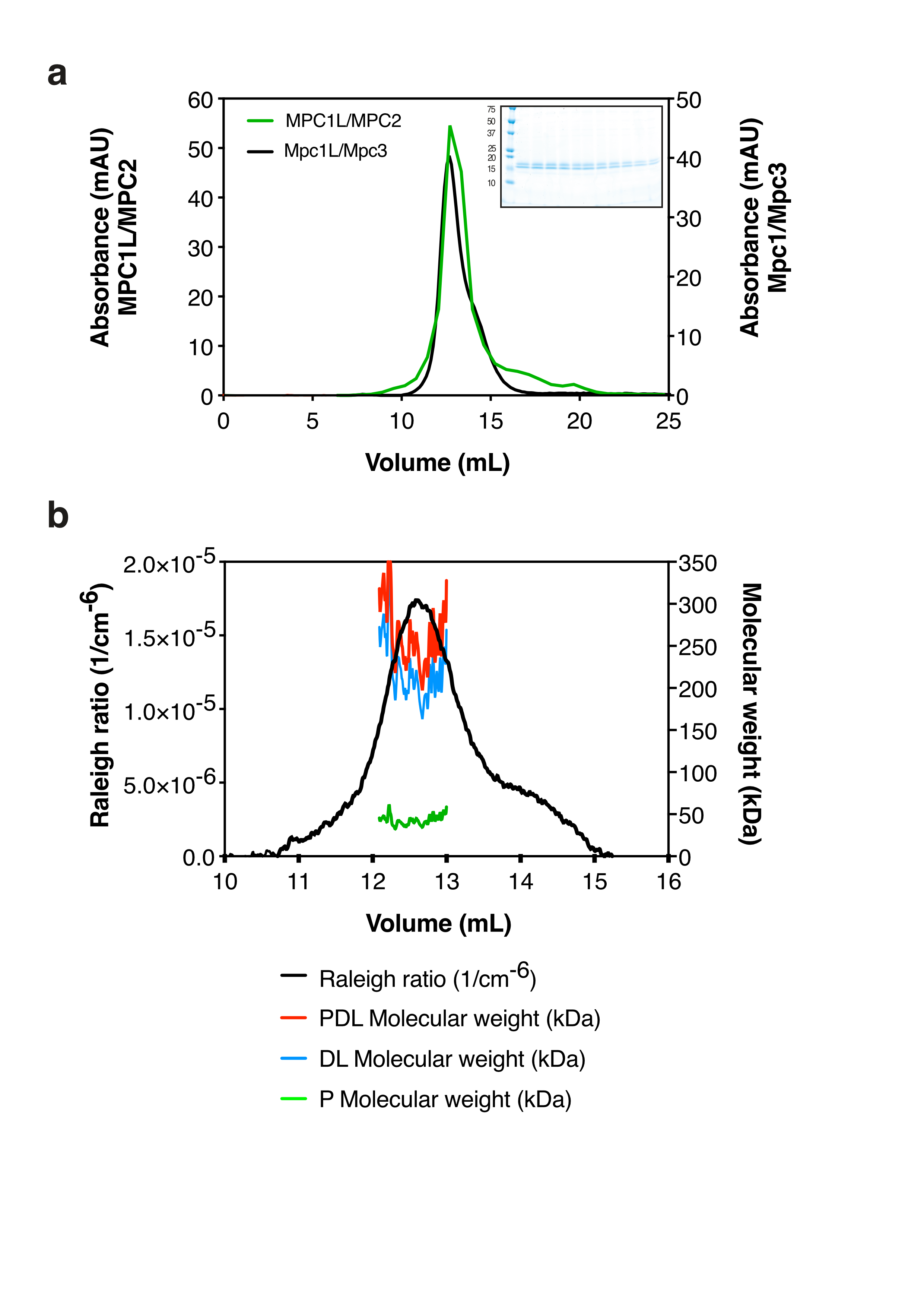


**Figure S2. Oligomeric state of human MPC1L/MPC2**

(**a**) Size-exclusion chromatography and superimposed A_280_ profiles for the Mpc1/Mpc3 hetero-dimer [4] (black) and MPC1L/MPC2 (green) purified via Nickel-affinity chromatography. *Inset:* Peak fractions for MPC1L/MPC2 analyzed by SDS-PAGE and visualized by Coomassie Blue stain.

(**b**) SEC-MALLS analysis of MPC1L/MPC2. The light scattering trace is shown as a black line. The masses of the protein-detergent-lipid complex (PDL), the detergent-lipid micelle (DL) and the protein (P) are indicated in red, blue and green, respectively.

**
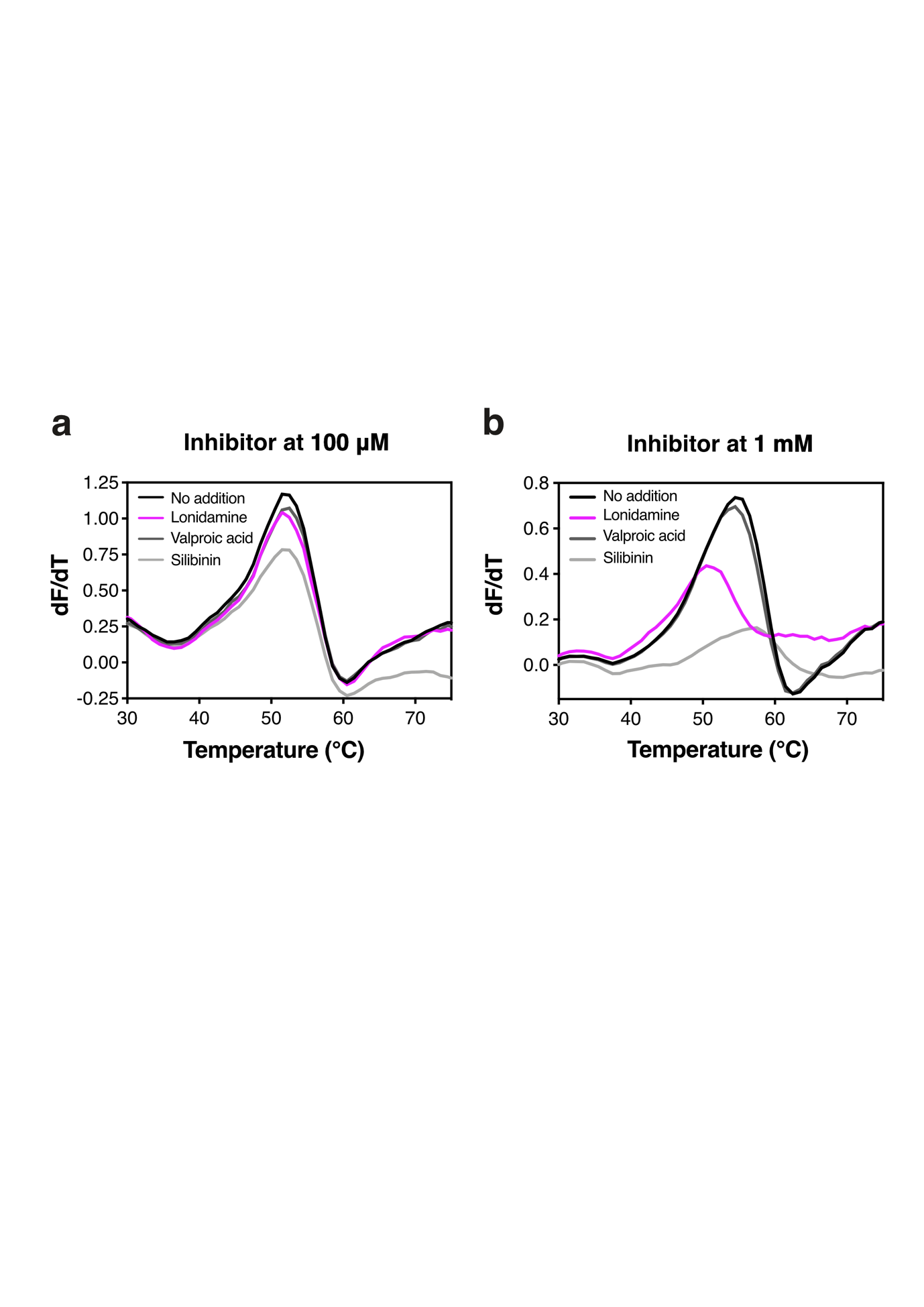
**

**Figure S3. Thermostability analysis of MPC in the presence of lonidamine, silibinin and valproic acid**

(**a**) First derivatives of protein unfolding curves obtained for MPC1L/MPC2 by the CPM thermostability shift assay at 100 μM, lonidamine, valproic acid or silibinin.

(**b**) First derivatives of protein unfolding curves obtained for MPC1L/MPC2 by the CPM thermostability shift assay at 1 mM lonidamine, valproic acid or silibinin.

The results are representative of two biological repeats, each performed in duplicate.

**
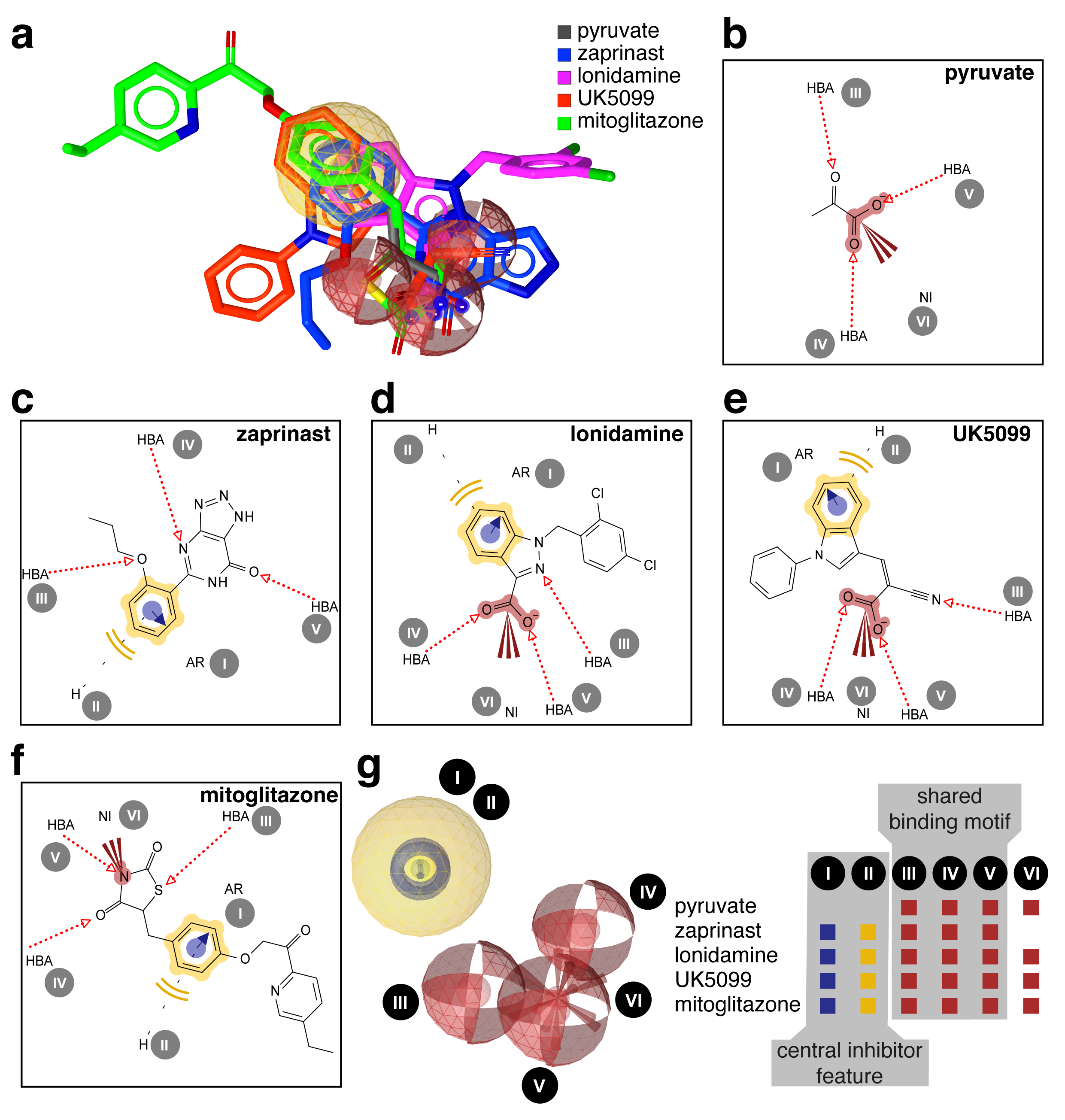
**

**Figure S4. Structurally diverse MPC inhibitors have common macromolecular interactions**

(**a**) Shared-feature pharmacophore model based on four prototypic MPC inhibitors and the substrate pyruvate.

(**b**) Individual 2D pharmacophore representation of pyruvate.

(**c**) Individual 2D pharmacophore representation of zaprinast,

(**d**) Individual 2D pharmacophore representation of lonidamine.

(**e**) Individual 2D pharmacophore representation of UK5099.

(**f**) Individual 2D pharmacophore representation of mitoglitazone.

(**g**) 3D representation of the pharmacophore with the identified features and their presence in the various prototypic MPC inhibitors.

(**a-g**) Red spheres indicate hydrogen bond acceptor features, yellow spheres hydrophobic ring features, red stars indicate negative ionizable features, and blue tori indicate aromatic ring features. Pharmacophore features are abbreviated as follows: HBA, hydrogen bond acceptor; NI, negative-ionizable; H, hydrophobic ring; AR, aromatic ring. All features are identified by roman numbers.

**
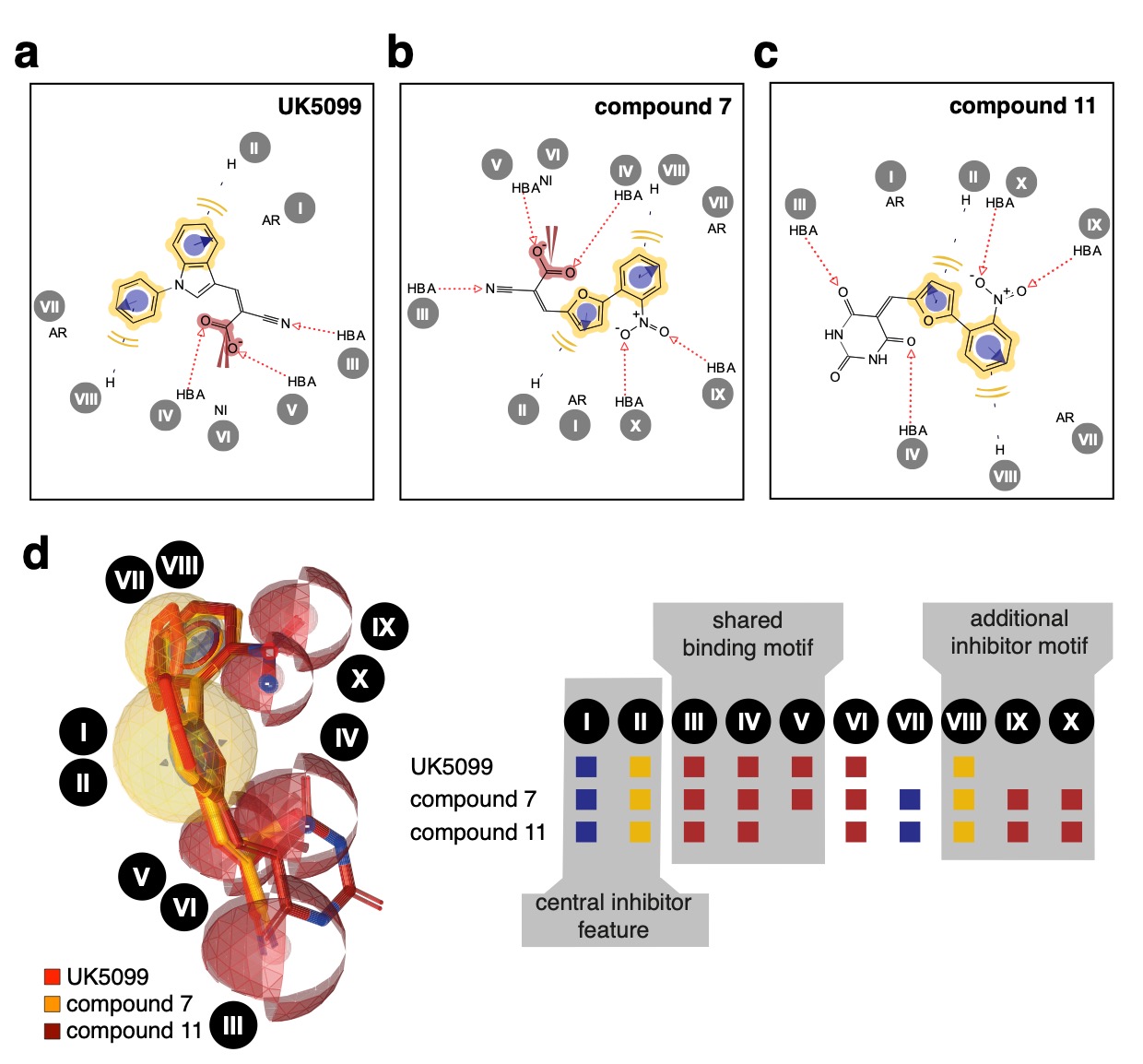
**

**Figure S5. Pharmacophore matching of UK5099, compound 7 and compound 11**

(**a**) 2D representation of pharmacophore properties for UK5099.

(**b**) 2D representation of pharmacophore properties for compound 7.

(**c**) 2D representation of pharmacophore properties for compound 11.

(**d**) Shared-feature pharmacophore model based on UK5099, compound 7 and compound 11.

Pharmacophore features are abbreviated as follows: HBA, hydrogen bond acceptor; NI, negative-ionizable; H, hydrophobic ring; AR, aromatic ring. All features are identified by roman numbers. Red spheres indicate hydrogen acceptor features, yellow spheres hydrophobic ring features, red stars indicate negative ionizable features, and bleu tori indicate aromatic ring features.

**
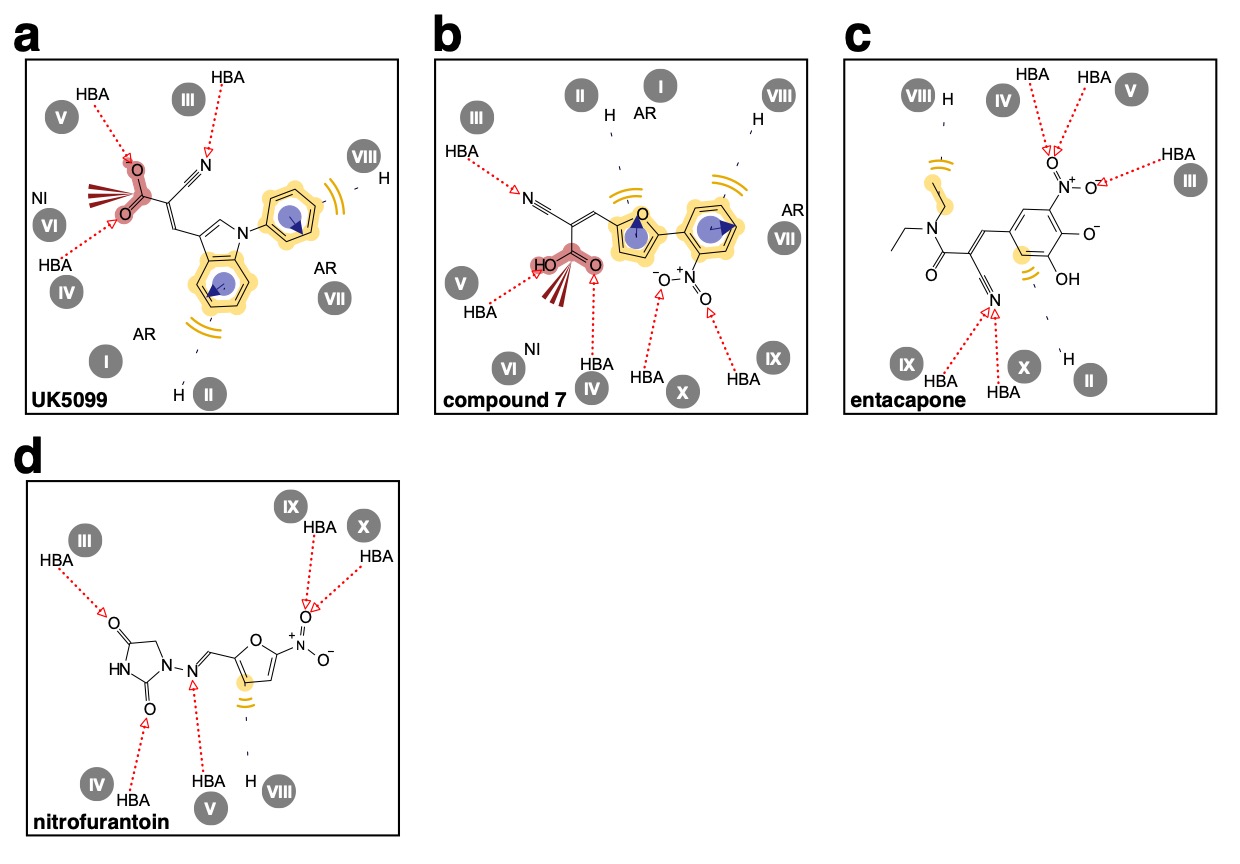
**

**Figure S6. 2D representation of pharmacophore properties for UK5099, compound 7, nitrofurantoin and entacapone**

(**a**) 2D representation of the pharmacophore properties for UK5099.

(**b**) 2D representation of the pharmacophore properties for compound 7.

(**c**) 2D representation of the pharmacophore properties for entacapone.

(**d**) 2D representation of the pharmacophore properties for nitrofurantoin.

Pharmacophore features are abbreviated as follows: HBA, hydrogen bond acceptor; NI, negative-ionizable; H, hydrophobic ring; AR, aromatic ring.

**Table S1.** Tm values obtained for the human MPC1L/MPC2 complex, via CPM and nano-DSF thermostability shift assays, in the presence or absence of inhibitors. Data represents the mean ± s.d. from 3 (CPM) or 2 (nano-DSF) biological repeats, each performed in duplicates or triplicates. *ND* stands for “not defined”.

| Compound | CPM | ∆Tm | Nano-DSF | ∆Tm |
| --- | --- | --- | --- | --- |
| No addition | 51.3 ± 0.2 | Not applicable | 51.9 ± 0.1 | Not applicable |
| UK5099 | 62.5 ± 0.9 | 11.2 ± 1.1 | 61.1 ± 0.6 | 9.2 ± 0.7 |
| CHC | 59.6 ± 0.4 | 8.3 ± 0.6 | 58.9 ± 0.4 | 7.0 ± 0.5 |
| Zaprinast | 58.8 ± 0.4 | 7.5 ± 0.6 | 58.2 ± 0.3 | 6.3 ± 0.4 |
| Lonidamine | 51.1 ± 0.3 | -0.2 ± 0.5 | 52.5 ± 0.2 | 0.6 ± 0.3 |
| Rosiglitazone | 56.6 ± 0.3 | 5.3 ± 0.5 | *ND* | *ND* |
| Pioglitazone | 56.4 ± 0.7 | 5.1 ± 0.9 | 53.0 ± 0.2 | 1.1 ± 0.3 |
| Mitoglitazone | 56.7 ± 0.6 | 5.4 ± 0.8 | 53.8 ± 0.4 | 1.9 ± 0.5 |
| Silibinin | 51.4 ± 0.4 | 0.1 ± 0.6 | 52.1 ± 0.2 | 0.2 ± 0.3 |
| Valproic acid | 51.3 ± 0.3 | 0.0 ± 0.5 | 51.8 ± 0.2 | -0.1 ± 0.3 |
| Entacapone | *ND* | *ND* | 58.2 ± 1.0 | 6.3 ± 1.1 |
| Nitrofurantoin | *ND* | *ND* | 54.7 ± 0.2 | 2.8 ± 0.3 |
| Compound 1 | 60.4 ± 1.0 | 9.1 ± 1.2 | *ND* | *ND* |
| Compound 2 | 63.4 ± 0.9 | 12.1 ± 1.1 | *ND* | *ND* |
| Compound 3 | 59.7 ± 1.1 | 8.4 ± 1.3 | *ND* | *ND* |
| Compound 4 | 60.4 ± 1.3 | 9.1 ± 1.5 | *ND* | *ND* |
| Compound 5 | 60.3 ± 1.4 | 9.0 ± 1.6 | *ND* | *ND* |
| Compound 6 | 59.8 ± 1.4 | 8.5 ± 1.6 | *ND* | *ND* |
| Compound 7 | 67.8 ± 1.2 | 16.5 ± 1.4 | *ND* | *ND* |
| Compound 8 | 62.3 ± 1.7 | 11.0 ± 1.9 | *ND* | *ND* |
| Compound 9 | 63.0 ± 1.8 | 11.7 ± 2.0 | *ND* | *ND* |
| Compound 10 | 51.2 ± 0.9 | -0.1 ± 1.1 | *ND* | *ND* |
| Compound 11 | 57.7 ± 2.1 | 6.4 ± 2.3 | *ND* | *ND* |
| Compound 12 | 60.4 ± 0.7 | 9.1 ± 0.9 | *ND* | *ND* |
| Compound 13 | 58.7 ± 1.7 | 7.4 ± 1.9 | *ND* | *ND* |

**Table S2.** Results of compound preparation for pharmacophore modelling, including energy minimization using the MMFF94 protocol and generation of molecular conformations using the iCon protocol.

| Compound | No of iterations energy minimalization | Initial energy (kcal/mol) | Minimal energy (kcal/mol) | No of  Conformations |
| --- | --- | --- | --- | --- |
| Pyruvate | 116 | 85.77 | 80.66 | 1 |
| UK5099 | 226 | 192.19 | 56.70 | 14 |
| Zaprinast | 515 | 57.73 | -22.04 | 20 |
| Lonidamine | 431 | 263.29 | 128.00 | 11 |
| Mitoglitazone | 576 | 149.26 | 93.44 | 105 |
| Entacapone | 462 | 105.99 | 34.33 | 44 |
| Nitrofurantoin | 202 | 273.70 | 55.86 | 4 |
| Compound 1 | 266 | 42.36 | 10.84 | 25 |
| Compound 2 | 634 | 101.30 | 3.51 | 20 |
| Compound 7 | 532 | 132.35 | 14.78 | 16 |
| Compound 11 | 270 | 21.42 | -97.69 | 10 |

**REFERENCES**

1. Sievers, F., Wilm, A., Dineen, D., Gibson, T. J., Karplus, K., Li, W., Lopez, R., McWilliam, H., Remmert, M., Soding, J., Thompson, J. D. & Higgins, D. G. (2011) Fast, scalable generation of high-quality protein multiple sequence alignments using Clustal Omega, *Mol Syst Biol.* **7**, 539.

2. Buchan, D. W., Minneci, F., Nugent, T. C., Bryson, K. & Jones, D. T. (2013) Scalable web services for the PSIPRED Protein Analysis Workbench, *Nucleic acids research.* **41**, W349-57.

3. Jones, D. T., Taylor, W. R. & Thornton, J. M. (1994) A model recognition approach to the prediction of all-helical membrane protein structure and topology, *Biochemistry.* **33**, 3038-49.

4. Tavoulari, S., Thangaratnarajah, C., Mavridou, V., Harbour, M. E., Martinou, J. C. & Kunji, E. R. (2019) The yeast mitochondrial pyruvate carrier is a hetero-dimer in its functional state, *The EMBO journal.* **38**.
